# G-Box Factors 14-3-3 Proteins Negatively Regulate *Cucumber Mosaic Virus* Infection Tolerance in *Arabidopsis*

**DOI:** 10.3390/plants14203147

**Published:** 2025-10-13

**Authors:** Shunkang Zhou, Dongwei Huang, Yaling Zhao, Zejie Xie, Sen Lu, Lijuan Xie, Qingqi Lin, Hua Qi

**Affiliations:** 1 Guangdong Laboratory for Lingnan Modern Agriculture, Guangdong Provincial Key Laboratory of Agricultural & Rural Pollution Abatement and Environmental Safety, College of Natural Resources and Environment, South China Agricultural University, Guangzhou 510642, China; 2 Guangxi Key Laboratory of Agro-Environment and Agric-Products Safety, College of Agriculture, Guangxi University, Nanning 530003, China

**Keywords:** CMV, 14-3-3 protein, hormone signaling, transcription factors, autophagy, *Arabidopsis thaliana*

## Abstract

*Cucumber mosaic virus* (CMV), a representative species of the genus *Cucumvirus* in the family *Bromoviridae*, is globally distributed and infects over 1200 monocot and dicot plants. 14-3-3 proteins serve as molecular adaptors that bind phosphorylated target proteins and play significant roles in multiple signaling pathways, including plant growth and development, hormone signaling, and responses to abiotic and biotic stimuli. Although an increasing body of evidence supports the prominent roles of 14-3-3 proteins in regulating plant immunity, their specific roles in plant responses to CMV infection remain unclear. Here, we demonstrate that *14-3-3λ* and *14-3-3κ* knockout *Arabidopsis* plants display enhanced tolerance to CMV infection, with significantly suppressed viral replication compared to wild-type (WT) plants. Additionally, we conducted transcriptomics analysis by comparing the CMV-infected *14-3-3λ 14-3-3κ* (*14-3-3λ/κ*) double mutant to the WT using RNA-seq. The KEGG (Kyoto Encyclopedia of Genes and Genomes) enrichment and differentially expressed gene (DEG) results mainly suggest that plant hormone signaling, transcription factor activity, and the autophagy pathway are significantly involved in 14-3-3-mediated CMV tolerance in *Arabidopsis*. This study reveals new functions and potential molecular mechanisms of 14-3-3 proteins in regulating plant response to CMV infection and provides valuable insights into agricultural production.

## 1. Introduction

*Cucumber mosaic virus* (CMV), a representative member of the genus *Cucumovirus* in the family *Bromoviridae*, includes three subgroups: IA, IB, and II. CMV is globally distributed and infects more than 100 plant families, 500 genera, and 1000 plant species, making it one of the most widespread and economically damaging plant viruses [1]. CMV severely affects key economic crops, including tomato, banana, legumes, and cucurbits. Notably, CMV has caused substantial economic losses in major tobacco-growing regions worldwide and is one of the most important plant viruses globally due to its exceptionally broad host range and severe pathogenicity. Moreover, its characteristic genomic features and multifunctional proteins establish it as a model system for studying plant–virus interactions [2].

The 14-3-3 proteins, initially named G-box factors 14-3-3 (GF14), are a family of conserved eukaryotic proteins acting as regulatory molecules. They directly bind specific phosphoserine/phosphothreonine motifs in target proteins, thereby modulating their activity, localization, or stability [3]. In plants, 14-3-3 proteins regulate many physiological processes and responses to biotic and abiotic stresses [4,5,6]. Crucially, 14-3-3 proteins respond to pathogen infection by interacting with defense proteins [7,8,9]. For example, during beet black scorch virus (BBSV) infection in *Nicotiana benthamiana* leaves, the MAPK cascade is activated. The viral coat protein (CP) interacts with Nb14-3-3a and disrupts the association between Nb14-3-3a and NbMAPKKKα in a dose-dependent manner, destabilizing NbMAPKKKα and compromising the NbMAPKKKα-mediated antiviral defense response [7].

Moreover, OsGF14B positively regulates panicle blast resistance, but negatively regulates leaf blast resistance [10], while OsGF14C may negatively regulate blast resistance by suppressing the expression of *OsGF14E* and *OsGF14F* [4,10,11,12]. Furthermore, a recent study showed that overexpression of *Nb14-3-3h* in *Nicotiana. benthamiana* suppresses potato virus Y (PVY) replication, whereas its silencing enhances PVY accumulation. Dimeric Nb14-3-3h inhibits PVY infection by sequestering the susceptibility factor TRANSLATIONALLY CONTROLLED TUMOR PROTEIN (NbTCTP) [13]. These studies demonstrate the significant roles of 14-3-3 proteins in pathogen responses, with distinct functions of different 14-3-3 isoforms highlighting their complexity in viral responses.

Numerous studies have demonstrated that 14-3-3 proteins regulate plant stress responses by mediating multiple phytohormone signaling pathways, such as brassinosteroid (BR), auxin (indole-3-acetic acid, IAA), abscisic acid (ABA), gibberellin (GA), and ethylene (ET) [14]. Specially, 14-3-3 proteins integrate ABA signaling to regulate salt and drought responses [4,9,15]. However, further investigation is needed to analyze the interaction between 14-3-3 proteins and phytohormone signaling in plant response to biotic stresses.

In plant immunity, At *14-3-3λ* interacts with RESISTANCE TO POWDERY MILDEW 8 (AtRPW8.2), an R receptor that mediates salicylic acid (SA)-dependent resistance to the biotrophic fungal pathogen *Golovinomyces* spp, where *At14-3-3λ* overexpression enhances fungal resistance [16]. Consistently, the GIBBERELLIC ACID INSENSITIVE (GAI), REPRESSOR of GAI (RGA) and SCARECROW (SCR) (GRAS) transcription factor SCARECROW LIKE 7 (OsSCL7), induced by *Magnaporthe* oryzae infection, positively regulates rice immunity by activating defense-related genes. The 14-3-3 protein OsGF14c interacts with and stabilizes OsSCL7, as evidenced by reduced OsSCL7 protein levels and enhanced susceptibility in *osgf14c* mutants [17]. Conversely, the whitefly (*Bemisia tabaci*) Bt14-3-3 protein suppresses the DNA binding of tobacco ABSCISIC ACID-INSENSITIVE 5-LIKE (NtABI5) to the *PLANT DEFENSIN J1-2* (*NtPDF1.2*) promoter, compromising plant defense and enhancing whitefly performance. Analogously, a homologous 14-3-3 effector protein from *Myzus persicae* enhances aphid fitness by disrupting NtABI5-mediated NtPDF1.2 activation [18]. These findings collectively illustrate that 14-3-3 proteins act as pivotal regulators of plant immunity by integrating with transcription factors.

Autophagy is a conserved pathway for the degradation of intracellular components in lysosomes or vacuoles by resident hydrolases across eukaryotic cells [19,20,21,22]. Other studies have demonstrated that autophagy plays both anti- and pro-pathogen roles during CaMV infection [23,24]. Furthermore, emerging evidence suggests that autophagy is likely involved in plant immunity by directly interacting with pathogen factors. The virulence factor βC1 of *Cotton leaf curl Multan virus* (CLCuMuV) induces plant autophagy by binding GLYCERALDEHYDE-3-PHOSPHATE DEHYDROGENASE (NbGAPC) and undergoes autophagic degradation through interaction with NbATG8f, thus reducing virulence [25,26]. Conversely, viruses like *Barley stripe mosaic virus* (BSMV) induce γb factor to reduce NbATG7–NbATG8 association, inhibiting autophagy and exacerbating the disease [27]. Thus, autophagy plays essential roles in plant–pathogen interactions through multiple mechanisms. Considering that 14-3-3 proteins are involved in regulating plant autophagy [22], the connections between 14-3-3s, autophagy, and virus resistance need to be studied.

Although 14-3-3 proteins are known to coordinate phytohormone signaling, transcription factors, and autophagy to regulate stress responses, their specific molecular mechanism in modulating plant responses to CMV infection remains unknown. In this study, we found that deletion of *14-3-3λ* and *14-3-3κ* displayed enhanced tolerance to CMV in *Arabidopsis* with significantly reduced *CP* gene expression and protein accumulation compared to the wild-type (WT) plants, suggesting that 14-3-3 proteins positively modulate CMV infection in *Arabidopsis*. Subsequent high-throughput transcriptomic analysis revealed that CMV infection upregulated 1628 and downregulated 947 genes in the WT. In contrast, the *14-3-3λ/κ* double-mutant exhibited upregulation of 2462 genes and downregulation of 1792 genes versus the pre-inoculation levels. Notably, genes related to transcription factors, phytohormone signaling, and autophagy were more significantly altered in the *14-3-3λ/κ* double-mutant compared to the WT, potentially conferring plant resistance to CMV. These findings demonstrate that 14-3-3 proteins promote viral replication during CMV infection, potentially by regulating the transcription factor, phytohormone, and autophagy signaling pathways. This study provides potential genetic resources and valuable references for enhancing virus resistance in plants.

## 2. Results

### 2.1. 14-3-3 Proteins Positively Regulate CMV Infection in Arabidopsis

**Figure 1:**
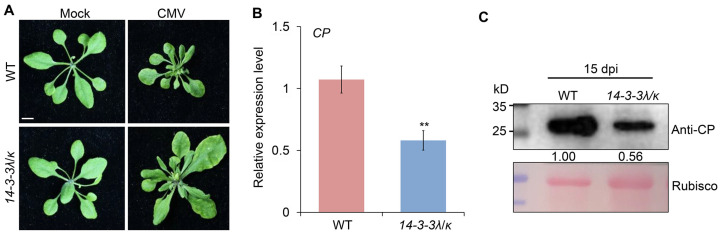
*14-3-3λ* and *14-3-3κ* negatively regulate *Cucumber mosaic virus* (CMV) infection in *Arabidopsis*. (**A**) Phenotype of wild-type (WT) and *14-3-3λ/κ* double mutant upon infection with CMV. Two-week-old WT and *14-3-3λ/κ* double mutant were inoculated with CMV for 15 days before photos were taken. Bar = 1 cm. (**B**) Relative transcription level of coat protein encoding gene (*CP*) in CMV-infected WT and *14-3-3λ/κ* double mutant at 15 days after inoculation. Values are means ± SD. ** *p* < 0.01 (*n* = 3, two-tailed Student’s *t*-test). (**C**) Analysis of CMV CP protein levels in leaves of WT and *14-3-3λ/κ* double-mutant line at 15 days after CMV inoculation. CMV CP proteins were detected by anti-CP antibodies (top panel). Ponceau S-stained membranes are shown below the blots to indicate the amount of protein loaded per lane (bottom panel). The relative intensity of the CP protein normalized to the loading control is shown below. Numbers on the left indicate molecular mass (kD) of size markers. dpi, days post-inoculation.

Previous studies have demonstrated that 14-3-3 proteins are key regulators of plant responses to many pathogens [7]. Specially, *14-3-3λ* and *14-3-3κ* work redundantly in the anti-bacterial and anti-fungal immunity of *Arabidopsis thaliana* [8]. To further investigate the potential cellular functions of *14-3-3λ* and *14-3-3κ* in CMV infection, we tested the responses of the *14-3-3λ/κ* double-mutant line [20] and WT plants to CMV inoculation. Phenotypic observation at 15 days post-inoculation (dpi) revealed obvious phenotypic differences between the *14-3-3λ/κ* double-mutant line and the WT (Figure 1A). Typically, the WT plants displayed abnormal leaf growth after CMV infection (Figure 1A). In contrast, the *14-3-3λ/κ* double-mutant plants exhibited few symptoms comparable to the mock-inoculated controls (Figure 1A). The altered leaf morphology, including leaf narrowing and deformation, observed in CMV-infected *14-3-3λ/κ* double-mutant plants, along with the relatively larger size of the first two leaves compared to WT, suggests slower symptom development and a potentially reduced susceptibility to CMV. To further quantify CMV accumulation, we performed RT-qPCR and Western blot assays on the CMV-infected *14-3-3λ/κ* double-mutant and WT plants. The results consistently demonstrated significantly reduced virus levels in the mutant compared to the WT. Specifically, RT-qPCR showed a substantial decrease in viral *CP* gene transcription in the *14-3-3λ/κ* double-mutant plants (Figure 1B). Correspondingly, Western blot analysis confirmed markedly lower levels of CP protein accumulation in the *14-3-3λ/κ* double mutant (Figure 1C). Collectively, these observations imply positive roles of 14-3-3 proteins in the modulation of CMV infection in plants.

### 2.2. RNA-Seq Data Processing

To elucidate the biological function of *Arabidopsis* 14-3-3 proteins in conferring resistance to CMV, we performed transcriptome profiling via RNA sequencing (Illumina platform) on the *14-3-3λ/κ* double-mutant (DM) and WT plants upon treatment with CMV and C buffer as a control (CK). Differentially expressed genes (DEGs) were identified through genotype (WT vs. DM) and treatment (CK vs. CMV) comparisons. The WT plants that were treated with C buffer (WT_CK samples) yielded 57,886,544, 39,148,298, and 43,172,010 raw reads. However, the *14-3-3λ/κ* double-mutant samples inoculated with C buffer (DM_CK samples) produced 43,448,062, 44,228,946, and 43,615,038 raw reads. Moreover, the WT samples upon inoculation with CMV (WT_CMV) generated 45,700,624, 42,499,218, and 43,226,060 raw reads. The *14-3-3λ/κ* double-mutant plants treated with CMV (DM_CMV) yielded 41,190,000; 42,983,302; and 42,112,378 raw reads (Figure S1).

### 2.3. Identification of DEGs in Arabidopsis Responding to CMV

**Figure 2:**
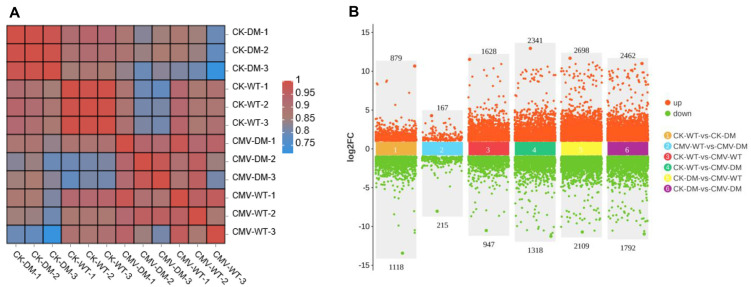
Analysis of gene expression in WT and *14-3-3λ/κ* double mutant (DM) after CMV inoculation. (**A**) Heatmap of WT and *14-3-3λ/κ* double-mutant plants under normal growth conditions (CK) or after inoculation with CMV for 15 days (CMV). The number following the sample name represents the number of biological replicates. (**B**) Number of differentially expressed genes (DEGs) in WT and *14-3-3λ/κ* double-mutant plants under normal growth conditions (CK) or after inoculation with CMV for 15 days (CMV). FC, fold change.

To comprehensively characterize the transcriptomic alterations underlying the differential responses of the *14-3-3λ/κ* double mutant and the WT to CMV infection in *Arabidopsis*, we identified the DEGs between the CMV-infected plants against their corresponding mock-inoculated controls (CK). The DEGs were stringently defined using established bioinformatic criteria, requiring an absolute log2 fold change (log2FC) ≥ 1 (equivalent to a 2-fold change in expression) and an adjusted *p*-value (FDR) < 0.05 to ensure statistical significance and minimize the false positives. The robustness of our transcriptional dataset was supported by the exceptionally high reproducibility observed among the triplicate biological replicates for each condition (*n* = 3). Furthermore, minimal intra-group variation, strong clustering of replicates, and clear separation were evident between the treatment groups, and the genotypes collectively confirmed the reliability and precision of the generated expression data (Figure 2A).

Our comparative transcriptomic analysis unveiled profound differences in the basal gene expression between the *14-3-3λ/κ* double-mutant and WT plants even under non-stressed (CK) conditions. In the absence of CMV infection, 1997 genes were differentially regulated in the *14-3-3λ/κ* double mutant relative to the WT, with 879 genes significantly upregulated and 1118 genes significantly downregulated (Figure 2B).

This baseline transcriptional difference highlights the fundamental rewiring of gene networks resulting from the loss of *14-3-3λ* and *14-3-3κ* functions. Strikingly, although the total number of DEGs was lower following infection, CMV inoculation dramatically altered the transcriptomic divergence between genotypes, revealing a strong and specific response in the *14-3-3λ/κ* double mutant that was not apparent under control conditions. Specifically, 1997 genes were differentially expressed in the infected double mutant compared to the infected WT, with 879 upregulated and 1118 downregulated. Following CMV inoculation, the number of DEGs specifically was 382 genes that are particularly responsive to viral infection, comprising 167 significantly upregulated and 215 significantly downregulated in the *14-3-3λ/κ* double mutant relative to the infected WT (Figure 2B). This pronounced shift in the global gene expression profile, characterized by a transition from widespread differential expression at the baseline to a more focused set of CMV infection-modulated DEGs, strongly implicates that *14-3-3λ* and *14-3-3κ* act as key regulators to orchestrate transcriptional programs during plant defense.

### 2.4. Gene Ontology (GO) and Kyoto Encyclopedia of Genes and Genome (KEGG) Analyses

**Figure 3:**
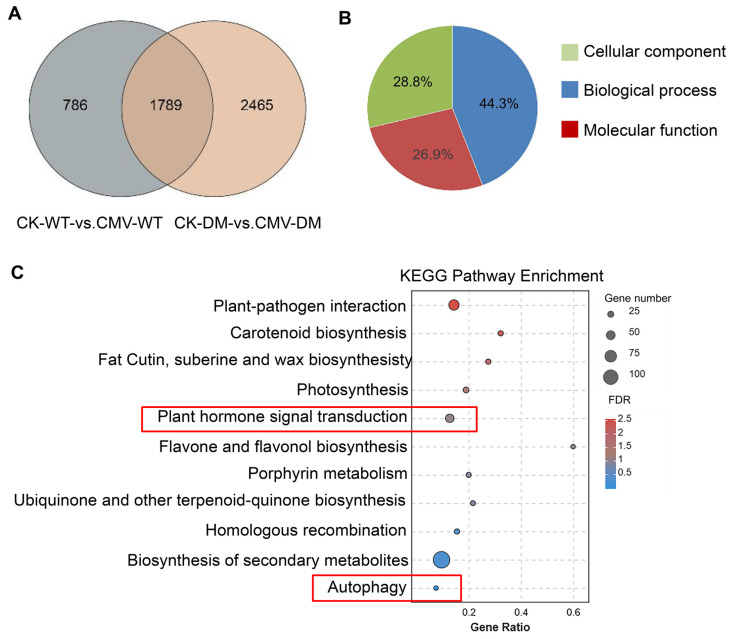
Gene expression patterns and KEGG enrichment analyses for CK-WT vs. CMV-WT and CK-DM vs. CMV-DM groups. (**A**) Venn diagram of DEGs identified in CK-WT vs. CMV-WT and CK-DM vs. CMV-DM groups. CK, control plant; CMV, plant inoculated with CMV; DM, *14-3-3λ/κ* double-mutant plant. (**B**) GO enrichment analysis of DEGs. Different colors represent different functional categories. (**C**) KEGG enrichment plot for DEGs in CK-WT vs. CMV-WT and CK-DM vs. CMV-DM groups. Enrichment significance was measured by the Rich factor, FDR value, and the number of genes enriched in the pathway. The Rich factor refers to the ratio of enriched differentially expressed genes in a pathway to total annotated differentially expressed genes. Higher values indicate greater enrichment. FDR values are in the range of 0–1; values closer to 0 indicate greater significance. KEGG, Kyoto Encyclopedia of Genes and Genomes.

To gain deeper insights into the molecular mechanisms underlying the altered CMV responses in the *14-3-3λ/κ* double mutant, we conducted comprehensive functional enrichment analysis of DEGs under control (CK) and CMV-infected conditions. We identified 1787 common differentially expressed genes in the CK-WT vs. CMV-WT and CK-DM vs. CMV-DM. There were 786 and 2465 specific differentially expressed genes detected in the comparisons of CK-WT vs. CMV-WT and CK-DM vs. CMV-DM, respectively (Figure 3A). Gene Ontology (GO) enrichment analysis of the 2465 *14-3-3λ/κ* double-mutant-associated DEGs revealed 53 significantly enriched terms (adjusted *p*-value < 0.05) compared to the whole *Arabidopsis* genome background. These enriched terms were distributed across three primary GO domains: Biological Processes (BPs) constituted the largest proportion at 44.3% (*n* = 24 terms), Molecular Functions (MFs) accounted for 26.9% (*n* = 14 terms), and Cellular Components (CCs) represented 28.8% (*n* = 15 terms) (Figure 3B). This distribution highlights the broad impact of *14-3-3λ/κ* deficiency on many cellular activities, ranging from dynamic processes to molecular interactions and subcellular localization.

Subsequent classification of the enriched GO terms focused on identifying the biologically relevant pathways. Using stringent hypergeometric testing, we selected the top 11 most significantly enriched GO terms within the target gene set for a focused investigation. Crucially, this prioritized list included terms directly linked to phytohormone and autophagy pathways (Figure 3C). Further analysis of the genes annotated to these key terms revealed that the *14-3-3λ/κ* double mutant exhibited higher enrichment density for genes in biosynthesis of secondary metabolites, plant–pathogen interaction, and plant hormone signal transduction (Figure 3C). Furthermore, consistent with the known regulatory connection between 14-3-3 proteins and autophagy, 13 autophagy-related genes were robustly enriched within the autophagy pathway (Figure 3C). These findings suggested that 14-3-3 proteins regulate CMV infection mainly by modulating secondary metabolite biosynthesis, plant hormone signaling, and the autophagy pathway.

### 2.5. Classification of Differentially Expressed Transcription Factors upon CMV Infection

**Figure 4:**
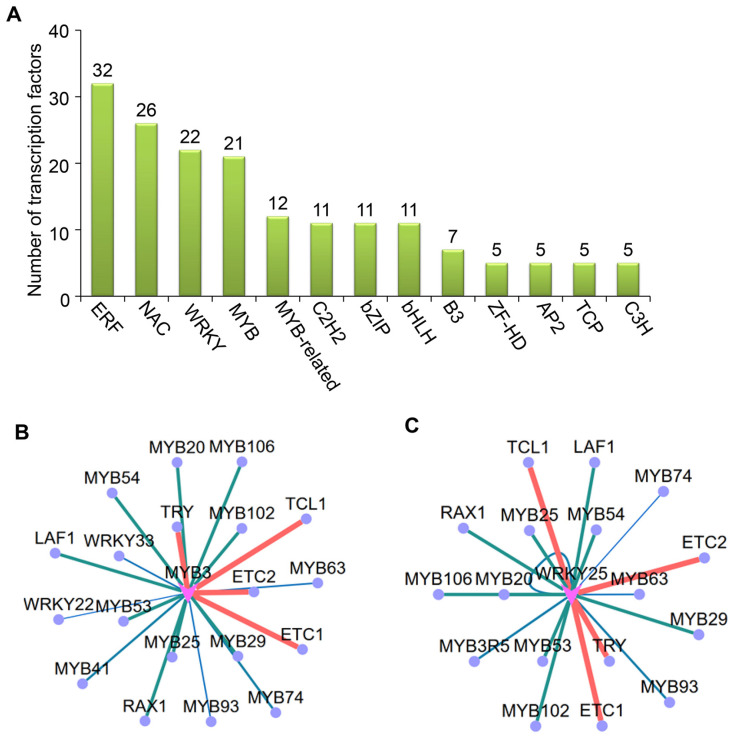
Analyses of differentially expressed transcription factors (TFs) in WT and *14-3-3λ/κ* double-mutant plants upon CMV infection for 15 days. (**A**) Classification of differentially expressed TFs (top 13). (**B**,**C**) Targeting analysis of MYB3 (**B**) and WRKY25 (**C**).

Based on the comprehensive transcriptome profiling data, we systematically identified the transcription factors (TFs) exhibiting significant differential expression following CMV infection. These differentially expressed TFs were meticulously annotated according to established family classifications, revealing the top 13 predominant TF families with the most remarkable expression alterations. A histogram illustrates the quantitative distribution of differentially expressed TFs across each family in the WT and *14-3-3λ/κ* double-mutant plants upon CMV infection. The results demonstrated that specific TF families exhibit substantial enrichment, notably ERF (ethylene response factor), NAC [NO APICALMERISTEM (NAM), ATAF1/2, and CUP-SHAPED COTYLEDON2 (CUC2)], WRKY, MYB (MYELOBLASTOSIS), and several other families implicated in stress adaptation mechanisms (Figure 4A). This aligns with the previous findings demonstrating that the ERF, NAC, WRKY, and MYB transcription factors play key regulatory roles in viral infection [28,29,30,31]. Particularly noteworthy is the altered expression pattern observed in the ERF, NAC, WRKY, and MYB families, suggesting that *14-3-3λ* and *14-3-3κ* likely regulate host immune responses through selective modulation of disease-resistance-associated TF families. This observation implies that *14-3-3λ/κ* serves as a regulatory nexus coordinating transcriptional reprogramming during viral challenge.

Building on these findings, we integrated the prediction of promoter binding motifs with co-expression network analysis to identify the potential downstream target gene groups of these differentially expressed TFs. This multi-omics strategy enabled systematic identification of potential downstream target genes governed by differentially expressed TFs. An interaction network diagram was constructed to visualize the regulatory relationships between the core TFs and their putative targets, where node size and color gradation represent the significance of expression change and regulatory intensity, respectively (Figure 4B). Our analysis revealed that hub TFs (represented as central network nodes) potentially orchestrate extensive transcriptional reprogramming of stress-responsive genes. Notably, the MYB and WRKY transcription factors exhibited targeted binding to their potential substrates following CMV infection (Figure 4B). Moreover, MYB3 and WRKY25 primarily modulate plant resistance to CMV by regulating other MYB or WRKY TFs, suggesting that functional coordination among distinct transcription factors is critical for 14-3-3 protein-mediated antiviral responses.

### 2.6. 14-3-3 Proteins Coordinate Phytohormone and Autophagy Signaling Pathways During CMV Infection

**Figure 5:**
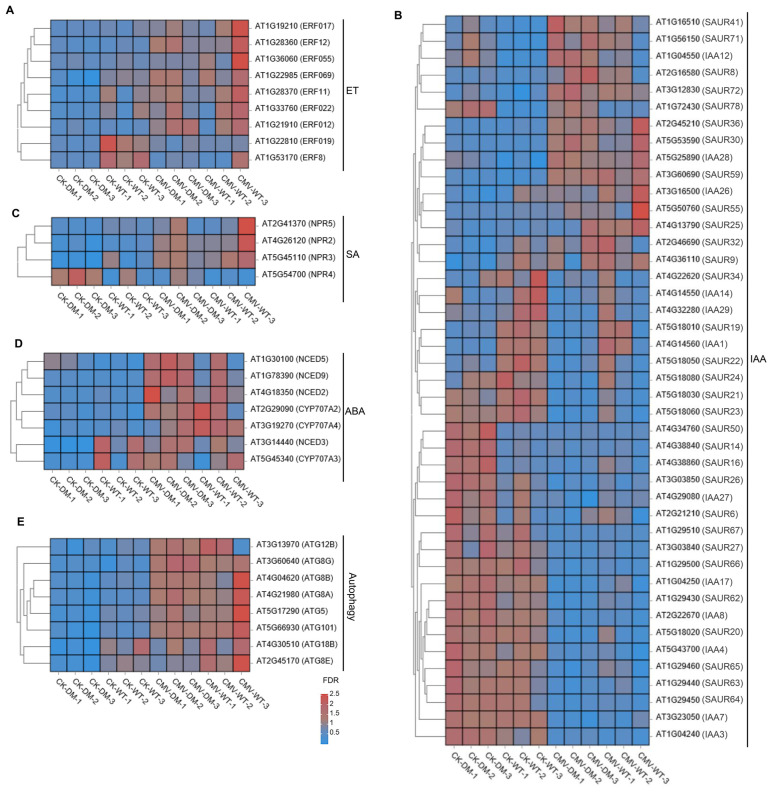
14-3-3 proteins coordinate phytohormones and autophagy signaling pathways to regulate CMV infection in *Arabidopsis*. Heatmap of DEGs in WT and *14-3-3λ/κ* double-mutant plants under normal growth conditions (CK) or infected with CMV for 15 days (CMV) for ET (**A**), IAA (**B**), SA (**C**), ABA (**D**), and autophagy (**E**) pathways. ET, ethylene; SA, salicylic acid; ABA, abscisic acid; IAA, indole-3-acetic acid.

An extensive body of evidence has demonstrated that phytohormone signaling networks constitute fundamental regulatory axes in plant stress adaptation. Notably, the ET signaling pathway has been specifically implicated in regulating the susceptibility to viral pathogens by modulating host transcriptional reprogramming [32,33,34]. Thus, we conducted systematic profiling of ET-associated transcriptional regulators and observed pronounced downregulation of these factors in the *14-3-3λ/κ* double-mutant plants compared to that of the WT upon CMV infection (Figure 5A). This transcriptional attenuation was associated with significantly reduced viral replication in the *14-3-3λ/κ* double-mutant line, suggesting that *14-3-3λ* and *14-3-3κ* may facilitate CMV infection through modulation of ET-mediated susceptibility mechanisms.

**Figure 6:**
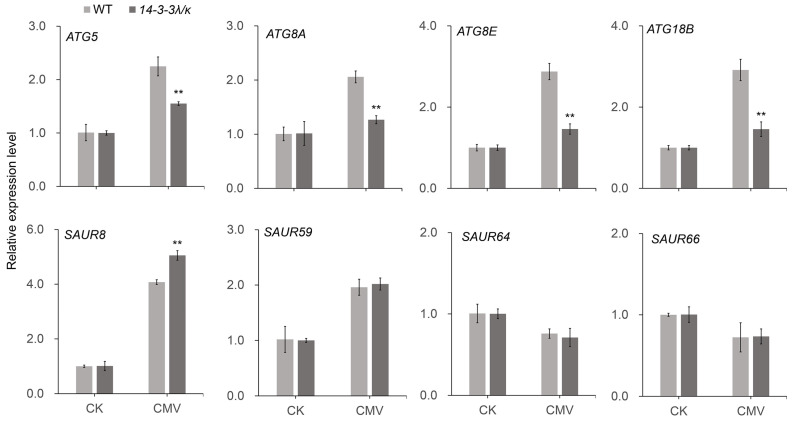
Expression analysis of autophagy-related and IAA-responsive genes. Relative expression levels of selected genes in the autophagy pathway (*ATG5*, *ATG8A*, *ATG8E*, *ATG18B*) and IAA signaling (*SAUR8*, *SAUR59*, *SAUR64*, *SAUR66*) in the WT and *14-3-3λ/κ* double-mutant plants under normal growth conditions (CK) or infected with CMV for 15 days (CMV). Data represent the mean ± SD (*n* = 3 biological replicates). Asterisks indicate statistically significant differences compared to the WT (** *p* < 0.01; Student’s *t*-test).

Comprehensive analysis of DEGs between the *14-3-3λ/κ* double mutant and the WT in response to CMV infection revealed that multiple phytohormone signaling pathways were involved in 14-3-3 protein-mediated tolerance. The most substantially altered genes correspond to core components of the IAA (indole-3-acetic acid) signaling cascade. Within this pathway, members of the SAUR (SMALL AUXIN UP RNA) family, key regulators of cell expansion and stress response, exhibit marked expression divergence between the *14-3-3λ/κ* double mutant and the WT. Here, *SAUR41*, *SAUR71*, *SAUR8*, *SAUR72*, and *SAUR78* are significantly upregulated; *SAUR19*, *SAUR21*, and *SAUR65* are downregulated in the *14-3-3λ/κ* double mutant compared to the WT (Figure 5B). Moreover, other *SAUR* family members, such as *SAUR36*, *SAUR22*, *SAUR24*, *SAUR21*, *SAUR23*, *SAUR50*, *SAUR14, SAUR67*, *SAUR27*, *SAUR66*, *SAUR62*, *SAUR63*, *SAUR59*, and *SAUR64*, exhibited minimal differential expression in the *14-3-3λ/κ* double mutant compared to the WT (Figure 5B). To further validate the RNA-seq data, we performed RT-qPCR on four randomly selected *SAUR* family genes (*SAUR8*, *SAUR59*, *SAUR64*, and *SAUR66*). The results showed that the expression pattern of these genes was in close agreement with the transcriptome data, thereby confirming the reliability of our RNA-seq analysis (Figure 6). Additionally, the *NONEXPRESSOR OF PATHOGENESIS-RELATED GENE* (*NPR*) family members are the key components of SA signaling and serve as central regulators in systemic acquired resistance in plants [35]. The observed differential expression patterns of *NPR* genes suggest a compromised SA-mediated defense response in the absence of functional *14-3-3λ* and *14-3-3κ* (Figure 5C). Furthermore, the key regulatory genes in ABA metabolism, such as *9-CIS-EPOXYCAROTENOID DIOXYGENASE* (*NCED*) and *CYTOCHROME P450*, FAMILY 707, SUBFAMILY A (*CYP707A*), two kinds of enzymes that critically regulate carotenoid-derived hormone precursors, displayed statistically significant expression shifts (Figure 5D). These findings imply potential cross-talk between ABA metabolism and 14-3-3-mediated antiviral responses in plants.

Intriguingly, our transcriptome profiling uncovered substantial modulation of core autophagy machinery components. *ATG5* (*AUTOPHAGY-RELATED 5*) and *ATG8* exhibited pronounced downregulation in the *14-3-3λ/κ* double mutant compared with the WT upon CMV infection (Figure 5E). This observation was further validated by RT-qPCR, which confirmed that although *ATG5*, *ATG8A*, *ATG8E*, and *ATG18B* were upregulated upon CMV infection in both the WT and the *14-3-3λ/κ* double-mutant plants, their expression levels remained significantly lower in the *14-3-3λ/κ double* mutant than in the WT (Figure 6). These results suggest decreased autophagic flux in *14-3-3λ/κ* double mutant compared to WT during CMV infection. As autophagy serves dual roles in viral defense, facilitating both viral clearance through xenophagy and supporting viral replication via membrane remodeling [36,37,38], these findings imply that *14-3-3λ* and *14-3-3κ* function as potential integrators of stress-induced autophagy. Collectively, these results demonstrate that *14-3-3λ* and *14-3-3κ* function as scaffold proteins to coordinate multiple signaling pathways involving phytohormone signaling and autophagy dynamics to regulate plant responses to CMV infection.

## 3. Discussion

14-3-3 proteins were first discovered in mammals and are widely distributed in eukaryotes [20]. Seven isoforms of these proteins, including τ, ε, β, γ, σ, η, and ζ, have been identified in mammals. In contrast, plants exhibit a greater diversity of 14-3-3 proteins [20, with 13 isoforms in *Arabidopsis* [20], 12 in tomato [39], 17 in tobacco, and 25 in banana [40]. Initially named G-box factor 14-3-3 (GF14) and encoded by *GENERAL REGULATORY FACTOR* (*GRF*) genes, 14-3-3 proteins specifically recognize and interact with phosphorylated target proteins, including protein kinases, phosphatases, transcription factors, and other functional proteins, to regulate their localization and stability. Through these interactions,14-3-3 proteins transduce signals and serve as molecular adaptors to modulate plant growth, development, and stress responses.

In this study, we demonstrated that the 14-3-3 proteins negatively regulate plant tolerance to CMV. Firstly, deletion of *14-3-3λ* and *14-3-3κ* in *Arabidopsis* resulted in enhanced tolerance to CMV inoculation compared to the WT (Figure 1A). Consistent with this phenotype, the transcription of the *CP* gene and accumulation of the CP protein were remarkably reduced in the *14-3-3λ/κ* double-mutant line relative to the WT (Figure 1B,C), indicating 14-3-3 proteins promote CMV replication in plants. Secondly, KEGG enrichment analysis and DEG profiling revealed that the plant hormone signal transduction, transcription factor activity, and autophagy pathways are significantly involved in 14-3-3-mediated CMV infection in *Arabidopsis* (Figure 2, Figure 3, Figure 4 and Figure 5). Collectively, these findings demonstrate the regulatory role of 14-3-3 proteins in plant CMV infection, providing potential new strategies to control CMV replication. However, investigations on the CMV infection development and DEGs of 14-3-3 overexpression lines would greatly enhance our understanding of their functional roles in modulating CMV infection.

In plant immune networks, 14-3-3 proteins serve as phosphorylation-dependent signaling hubs that precisely coordinate defense gene expression by specific binding to immunity-related transcription factors, regulating their stability, subcellular localization, and DNA-binding capacity. For instance, the rice 14-3-3 protein OsGF14c interacts with the GRAS family transcription factor OsSCL7, inhibiting its ubiquitination-mediated degradation. This interaction significantly enhances OsSCL7 protein accumulation, thereby activating defense gene expression during *Magnaporthe oryzae* infection. Genetic knockout of *OsGF14c* accelerates OsSCL7 degradation and markedly increases rice susceptibility to blast disease [17]. Similarly, At14-3-3 proteins recruit histone acetyltransferases to TGA transcription factor complexes, remodeling chromatin conformation of SA pathway genes (e.g., *NPR1*) to potentiate systemic acquired resistance (SAR). In contrast, the histone demethylase JMJ16 indirectly modulates the immunity-development balance by reducing the H3K4me3 levels at senescence-associated WRKY53 loci, thereby delaying defense-related programmed cell death [41].

Consistently, in this study, our results revealed the top 13 predominantly differentially expressed TFs in the WT and *14-3-3λ/κ* double-mutant plants upon CMV infection, notably including ERF, NAC, WRKY, MYB, and several other families implicated in stress adaptation mechanisms (Figure 4A). Although MYB and WRKY transcription factors have been extensively characterized in various plant stress responses, our study provides novelty by integrating transcriptome profiling, promoter motif analysis, and co-expression networks to systematically identify the potential downstream regulators modulated by these TFs specifically during CMV infection. Furthermore, we uncovered that MYB3 and WRKY25 coordinate with other MYB and WRKY family members to orchestrate antiviral defenses, thereby revealing a previously uncharacterized functional network mediated by 14-3-3 proteins. These findings suggest that *14-3-3λ* and *14-3-3κ* likely regulate host immune responses through selective modulation of disease-resistance-associated TF families.

Generally, 14-3-3 proteins function through protein–protein interactions in many signaling pathways. In this study, although we did not directly measure protein-level changes or physical interactions, the phenotypic and transcriptional alterations observed in the *14-3-3λ/κ* double mutant provide compelling indirect evidence supporting their role as negative regulators of defense against CMV. Specifically, the reduced viral coat protein accumulation, coupled with significant transcriptional reprogramming affecting stress-responsive, hormone-related, and transcription factor genes, consistently indicates that the absence of these 14-3-3 isoforms enhances antiviral responses. These findings align with established models in which 14-3-3 proteins modulate defense pathways through interaction with phosphorylated client proteins. Although previous studies have demonstrated that multiple CMV proteins can be phosphorylated [42,43], the functional connection **between 14-3-3 proteins** and these phosphorylated CMV proteins during CMV infection remains to be fully elucidated.

Plant hormone pathways are generally considered to play significant roles in pathogen response [33,34,44,45]. Recent studies have demonstrated that 14-3-3 proteins are involved in modulating plant hormone signaling in plants [34,46]. Ethylene, a critical phytohormone, strongly regulates plant growth and development. CONSTITUTIVE TRIPLE RESPONSE 1 (CTR1) kinase is a key component in the ethylene pathway, which acts as a negative regulator of signaling in plants [47,48]. Sequence alignment analysis revealed 41% similarity between the CTR1 and Raf kinase, a target of 14-3-3. The interaction between 14-3-3 and CTR1 is essential for ethylene signaling transduction in *Arabidopsis* [49]. Furthermore, 14-3-3 proteins negatively regulate gibberellin (GA) biosynthesis by preventing the nuclear translocation of the RSG (REPRESSION OF SHOOT GROWTH) transcription factor, a key GA biosynthesis regulator. This inhibits transcription of genes involved in GA synthesis, controlling the endogenous GA levels in plants [50].

In this study, DEG analysis revealed that the *14-3-3λ/κ* double mutant exhibited higher enrichment density for genes in plant hormone signal transduction (Figure 3C). For instance, we identified four and seven DEGs associated with the SA and ABA signaling pathways, respectively (Figure 5). Most notably, DEGs related to the IAA signaling pathway represented the largest subset, with a total of 43 genes showing differential expression, as shown in Figure 5. These findings collectively suggest that 14-3-3 proteins may regulate the CMV resistance through cross-talk with multiple kinds of plant hormones.

Autophagy is a conserved process to target intracellular components to vacuoles for degradation, which plays dual roles in plant–virus interactions by facilitating both viral clearance through xenophagy and supporting viral replication via membrane remodeling [23,25,26,27]. Moreover, 14-3-3 proteins enhance the association between SINATs and phosphorylated ATG13a, promoting their degradation via the SINATs-mediated 26S proteasome pathway. Furthermore, the interaction of 14-3-3 with phosphorylated ATG13a disrupts ATG1–ATG13 complex formation, thereby inhibiting autophagy [22]. These findings suggest the negative role of 14-3-3 protein in regulating plant autophagy. In this study, we found that *ATG5* and *ATG8* exhibited more pronounced downregulation in the *14-3-3λ/κ* double mutant than in the WT upon CMV infection (Figure 5E), suggesting potential disruption of autophagic flux. Collectively, all these observations demonstrate that 14-3-3 and autophagy are tightly linked to CMV infection in plants.

## 4. Conclusions and Prospects

In this study, our findings reveal a negative regulatory role of 14-3-3 proteins in CMV tolerance in *Arabidopsis*. Specifically, deletion of *14-3-3λ* and *14-3-3κ* in *Arabidopsis* exhibits enhanced CMV tolerance, with significantly suppressed viral replication compared to the WT. Transcriptomics indicates that plant hormone signaling, transcription factor activity, and autophagy pathways are involved in 14-3-3-mediated CMV infection. This study reveals novel 14-3-3 functions in regulating CMV infection and provides key mechanistic insights for CMV control in agriculture.

Although recent studies have provided valuable insights into the biological function of 14-3-3 proteins in regulating plant response to CMV infection [4], significant knowledge gaps regarding the underlying mechanisms persist. Future research should prioritize field testing of exogenous 14-3-3 proteins and explore the interactions among plant signaling pathways.

## 5. Materials and Methods

### 5.1. Plant Growth Conditions and Virus Infection

The Columbia (Col-0) accession served as the wild type. The *14-3-3λ/κ* double mutant has been described previously [20]. All seeds of *Arabidopsis thaliana* and *Nicotiana benthamiana* were surface-sterilized for 15 min in 20% (*v/v*) bleach solution containing 0.05% (*v*/*v*) Tween 20, and then rinsed with sterilized water at least five times. The *Arabidopsis thaliana* seeds were subsequently sown on a Murashige and Skoog (MS) agar medium (0.8%, *w*/*v*) supplemented with 1% (*w*/*v*) sucrose. After a three-day stratification period in the dark at 4 °C, the plates were subjected to long-day (LD; 16 h light/8 h dark) conditions at 22 °C using fluorescent bulbs (PHILIPS, TL5 21W/865) with a light intensity of 120–150 μmol m^−2^ s^−1^. One week post germination, the *Arabidopsis* seedlings were transferred to soil under either LD or short-day (SD; 8 h light/16 h dark) conditions for further growth and analysis. The tobacco seedlings were grown in soil in a greenhouse at 23 °C under a 12 h light/12 h dark photocycle. The virus used in this study was the Fny strain of *Cucumber mosaic virus* (CMV). *Nicotiana benthamiana* plants were used as the propagative host for CMV. CMV virions were diluted in buffer C (0.5 mM Na_2_EDTA, 5 mM sodium borate, and pH 9.0) to the required concentrations. Three fully expanded leaves of four-week-old *Arabidopsis* seedlings were inoculated with CMV (10 ng/μL) [51]. Leaves were collected at 3–4 days post-inoculation (dpi) for local infection analysis, and systemically infected leaves were harvested at the indicated time points for subsequent viral protein and RNA assays.

### 5.2. RNA Extraction

Total RNA was isolated from 0.1 g of CMV-infected *14-3-3λ/κ* double-mutant and wild-type (WT) plant leaf tissues using the HiPure Plant RNA Mini Kit (Vazyme, Nanjing, China) according to the manufacturer’s instructions. The C buffer-treated *14-3-3λ/κ* double-mutant line and WT plants were used as the control.

### 5.3. Quality Control, Alignment of Sequencing Data, and cDNA Library Construction

The enriched mRNA samples were fragmented into short segments using the fragmentation buffer, and subsequently reverse-transcribed into cDNA using the NEBNext Ultra RNA Library Prep Kit from Illumina (NEB #7530, New England Biolabs, Ipswich, MA, USA). Following purification, the double-stranded cDNA fragments underwent end repair, A-tailing, and Illumina adapter ligation. Poly(A) mRNA was enriched during library preparation using IGT^®^ Enzyme Plus Library Prep Kit V3 with oligo(dT) magnetic beads according to the manufacturer’s protocol. For transcriptome sequencing analysis, three biological replicates each of Arabidopsis healthy or CMV-infected plant leaf tissue were prepared. Subsequent cDNA libraries were constructed, and sequencing analysis was performed by the Jidiao Biotechnology Company, Guangzhou, China. The ligation products were then purified using AMPure XP Beads (1.0×) and subjected to PCR amplification. The final cDNA libraries were sequenced on an Illumina Novaseq6000 platform from the Gene Denovo Biotechnology Co. (Guangzhou, China).

Subsequently, to obtain high-quality clean reads, the raw sequencing data were processed using fastp (v0.18.0) with stringent filtering criteria: removal of poly-N sequences and adapter contamination, exclusion of reads containing more than 10% unknown nucleotides (Ns), and elimination of reads with over 50% low-quality bases (Q-value ≤ 20). The filtered data were then evaluated for quality metrics, including Q20 and Q30 scores and GC content. The clean reads were subsequently aligned to the *Arabidopsis thaliana* reference genome (TAIR10 version) under the default parameters. Transcript assembly and expression quantification were performed using StringTie (v1.3.1) with the reference-based approach. To quantify gene expression levels within each sample, FPKMs (Fragments Per Kilobase per Million mapped reads) values were calculated using RSEM. For cross-sample comparative analysis, differential expression was assessed using the DESeq2 package in R, based on raw read counts. This approach applies the median-of-ratios method for normalization and provides statistical testing for identifying differentially expressed genes in different samples.

### 5.4. RNA-Quality Assessment

To ensure analytical reliability of transcriptome profiling, the raw reads were rigorously processed using fastp (v0.18.0) to remove low-quality bases, adapter sequences, and short reads. This yielded high-quality clean reads. WT_CK retained 57,585,924; 38,962,372; and 42,983,240 reads. DM_CK retained 43,269,648; 44,057,490; and 43,425,964 reads. WT_CMV retained 45,517,780; 42,345,128; and 43,055,670 reads. DM_CMV retained 41,015,612; 42,836,724; and 41,963,400 reads (Figure S1). Data retention efficiency exceeded 99% across all the samples, with the Q20 scores consistently > 98% (Figure S1), confirming exceptional sequence quality suitable for robust DEG analysis.

### 5.5. Screening and Functional Enrichment Analysis of Differentially Expressed Genes

Differentially expressed genes (DEGs) were identified through comparative transcriptome analysis using stringent selection criteria (absolute fold-change ≥ 2 and FDR-adjusted *p*-value < 0.05). To further characterize these transcriptional alterations, functional enrichment analyses were performed to elucidate their underlying biological significance.

### 5.6. Real-Time Quantitative PCR (RT-qPCR) Analysis

For the RT-qPCR assay, 2 μg of total RNA was treated with DNase I for 10 min at 37 °C, and then the first-strand cDNA was synthesized using the HiScript II Q Select RT Super Mix with gDNA Wiper (Vazyme, Nanjing, China). RT-qPCR was performed on a CFX 96 Real-Time PCR Detection System (BioRad, Hercules, CA, USA) using the Taq Pro Universal SYBR qPCR Master Mix (Q712-02/03, Vazyme). *ACTIN2* was used as an internal control for analyzing the expression levels of the target genes. The relative gene expression levels were analyzed by the 2^−∆∆CT^ method. All the reactions were performed, with three biological and technical replicates. The gene-specific primers used for RT-qPCR analysis are shown in Table S1.

### 5.7. Protein Isolation and Immunoblotting Assay

Protein isolation was performed as described previously [21]. The total proteins were extracted by lysis buffer (50 mM sodium phosphate [pH 7.0], 200 mM NaCl, 10 mM MgCl_2_, 0.2% β-mercaptoethanol, and 10% glycerol) supplemented with protease inhibitor cocktail (Roche 04693132001). The mixture was incubated on ice for 30 min and centrifuged at 4 °C for 30 min at 12,000× *g*. The resulting supernatant was transferred to a new microfuge tube containing 5 × sodium dodecyl sulfate (SDS) loading buffer and denatured at 95 °C for 10 min before electrophoresis.

For immunoblotting analysis, the denatured total proteins were separated by 10% SDS-PAGE, and subsequently transferred to a nitrocellulose (NC) membrane. Then, the NC membranes were blocked with 5% (*w*/*v*) non-fatted milk powder dissolved in 20 mL 1 × TBST buffer [20 mM Tris-HCl (pH 7.5), 150 mM NaCl, 0.05% (*v*/*v*) Tween-20] for 1 h at room temperature. All the primary antibodies listed below were added to 10 mL TBST buffer at a different ratio by incubation at 37 °C for 1 h. Anti-CP (1:3000) antibodies were used to detect the CP proteins.

### 5.8. Statistical Analysis

The data in this study are presented as the standard deviation (SD) ± from three independent experiments. A two-tailed Student’s *t*-test was employed for statistical analysis to assess the differences between the groups. A significance level of *p* < 0.05 or <0.01 is considered to be statistically significant. Pearson’s correlation coefficient for each gene pair was calculated across all samples. The resulting *p*-values were adjusted for multiple comparisons using the Benjamini–Hochberg false discovery rate (FDR) method. A correlation was considered statistically significant if the absolute coefficient |r| > 0.7 and an FDR-adjusted *p*-value < 0.05.

## Data Availability

The original contributions presented in this study are included in this article. Further inquiries can be directed to the corresponding author.

## Supplementary Materials

The following supporting information can be downloaded at: https://www.mdpi.com/article/10.3390/plants14203147/s1, Figure S1: Statistical table of assembly results. Table S1: Sequence of Primers Used in This Study.
