# Supplementary material for: G-Box Factors 14-3-3 Proteins Negatively Regulate Cucumber Mosaic Virus Infection Tolerance in Arabidopsis
Source: Plants (Basel). 2025 Oct 13;14(20):3147. doi: 10.3390/plants14203147 (PMC12567101; doi:10.3390/plants14203147)
Supplement: Supplementary file 1 [file plants-14-03147-s001.zip › plants-3848331-supplementary.pdf]

Sample	Raw Data	Clean Data (%)	Raw Data (bp)	After filter Q20 (%)	GC (%)
CK-WT-1	57886544	57585924 (99.48%)	6517209300	6386250908 (98.98)	2978622840 (46.17)
CK-WT-2	39148298	38962372 (99.53%)	6634341900	6498956802 (99.02)	3018755557 (46.00)
CK-WT-3	43172010	42983240 (99.56%)	6542255700	6402413968 (98.95)	2989109116 (46.19)
CMV-WT-1	45700624	45517780 (99.60%)	8682981600	8477655623 (98.88)	3962825531 (46.22)
CMV-WT-2	42499218	42345128 (99.64%)	5872244700	5742105384 (98.90)	2693345381 (46.39)
CMV-WT-3	43226060	43055670 (99.61%)	6475801500	6337246213 (98.95)	2963415235 (46.27)
CK-DM-1	43448062	43269648 (99.59%)	6178500000	6055812657 (98.94)	2812681576 (45.96)
CK-DM-2	44228946	44057490 (99.61%)	6447495300	6320986996 (98.94)	2918726432 (45.68)
CK-DM-3	43615038	43425964 (99.57%)	6316856700	6188280898 (98.90)	2856114339 (45.65)
CMV-DM-1	41190000	41015612 (99.58%)	6855093600	6710163852 (98.94)	3125623139 (46.09)
CMV-DM-2	42983302	42836724 (99.66%)	6374882700	6249414051 (98.99)	2894230311 (45.85)
CMV-DM-3	42112378	41963400 (99.65%)	6483909000	6351712502 (98.92)	2952755807 (45.99)

Figure S1. Statistical table of assembly results.
CK-WT, wild-type (WT) Arabidopsis grown under normal growth conditions; CK-DM: *14-3-3λ/κ* double mutant grown under normal growth conditions; CMV-WT, WT Arabidopsis upon CMV inoculation for 15 days; CMV-DM, *14-3-3λ/κ* double mutant plants after CMV inoculation for 15 days. The numbers following the sample name represent intra group duplications.

Table S1. Sequence of Primers Used in This Study.

Gene	Name	Sequence (5'→3')	Usage
ACTIN2	ML1124	CCCGCTATGTATGTCGC	RT-qPCR
	ML1125	AAGGTCAAGACGGAGGAT	
CMV CP	CMV CP-F	CCTCCTCCGCGGATGCTAAC	RT-qPCR
	CMV CP-R	TGGACGACCAGCTGCTAACG	
ATG5	ATG5-F	ACTGATACCATGTGAAGGAG	RT-qPCR
	ATG5-R	GTATAGGCATCAAGATCACC	
ATG8A	ATG8A-F	GCAGAGACTAATCGAATCGC	RT-qPCR
	ATG8A-R	CAAGCAACGGTAAGAGATCC	
ATG8E	ATG8E-F	CCCTGATCGAATTCCTGTGA	RT-qPCR
	ATG8E-R	GGAAGCCATCTTCGTCTTTC;	
ATG18B	ATG18B-F	CTACTTGCAATTGTTGGAGC	RT-qPCR
	ATG18B-R	CTGGAACAGCTAAGTAGCAG	
SAUR8	SAUR8-F	CAAGCTTGGGGAAGAAGAAT	RT-qPCR
	SAUR8-R	GGAATGGTGAGACCCATGTC	
SAUR59	SAUR59-F	TTTGCAAGCCGAGAGCCATC	RT-qPCR
	SAUR59-R	ACTCTCTGAAAGTCGCCGTC	
SAUR64	SAUR64-F	GCAACAAAGAGCAGCCCTCC	RT-qPCR
	SAUR64-R	GGCGATGTGATAGGTCCACC	
SAUR66	SAUR66-F	GGCAACAGAGAGCAGCTCTC;	RT-qPCR
	SAUR66-R	TGTGATTGGTCCACCGGTTG	
